# Anaerobic degradation of cyclohexane by sulfate-reducing bacteria from hydrocarbon-contaminated marine sediments

**DOI:** 10.3389/fmicb.2015.00116

**Published:** 2015-02-20

**Authors:** Ulrike Jaekel, Johannes Zedelius, Heinz Wilkes, Florin Musat

**Affiliations:** ^1^Department of Microbiology, Max Planck Institute for Marine MicrobiologyBremen, Germany; ^2^Organic Geochemistry, Helmholtz Centre Potsdam GFZ German Research Centre for GeosciencesPotsdam, Germany; ^3^Isotope Biogeochemistry, Helmholtz Centre for Environmental Research - UFZLeipzig, Germany

**Keywords:** cyclohexane, anaerobic, marine sediments, sulfate-reducing bacteria, *Desulfosarcina*

## Abstract

The fate of cyclohexane, often used as a model compound for the biodegradation of cyclic alkanes due to its abundance in crude oils, in anoxic marine sediments has been poorly investigated. In the present study, we obtained an enrichment culture of cyclohexane-degrading sulfate-reducing bacteria from hydrocarbon-contaminated intertidal marine sediments. Microscopic analyses showed an apparent dominance by oval cells of 1.5 × 0.8 μm. Analysis of a 16S rRNA gene library, followed by whole-cell hybridization with group- and sequence-specific oligonucleotide probes showed that these cells belonged to a single phylotype, and were accounting for more than 80% of the total cell number. The dominant phylotype, affiliated with the *Desulfosarcina-Desulfococcus* cluster of the Deltaproteobacteria, is proposed to be responsible for the degradation of cyclohexane. Quantitative growth experiments showed that cyclohexane degradation was coupled with the stoichiometric reduction of sulfate to sulfide. Substrate response tests corroborated with hybridization with a sequence-specific oligonucleotide probe suggested that the dominant phylotype apparently was able to degrade other cyclic and *n*-alkanes, including the gaseous alkane *n*-butane. Based on GC-MS analyses of culture extracts cyclohexylsuccinate was identified as a metabolite, indicating an activation of cyclohexane by addition to fumarate. Other metabolites detected were 3-cyclohexylpropionate and cyclohexanecarboxylate providing evidence that the overall degradation pathway of cyclohexane under anoxic conditions is analogous to that of *n*-alkanes.

## Introduction

Cycloalkanes are major constituents of crude oils, accounting for 20–40% of the total hydrocarbon fractions (Tissot and Welte, 1984), and are common in refined petroleum products. Cyclopentane, cyclohexane and their alkylated derivatives, especially methylcyclopentane and methylcyclohexane are most abundant (Tissot and Welte, 1984). Due to their wide industrial use as solvents and raw materials in the chemical industry, cycloalkanes are common environmental contaminants. Biodegradation studies of oil spills in ocean surface waters have shown that cycloalkanes were degraded to a lesser extent than *n*-alkanes (Brown and Huffman, 1976; Leahy and Colwell, 1990). This recalcitrance toward biodegradation can be in part attributed to the higher toxicity of cyclic alkanes as compared to *n*-alkanes (Sikkema et al., 1994, 1995). Many studies of cycloalkane biodegradation used cyclohexane as a model compound (Stirling et al., 1977; Anderson et al., 1980; Trower et al., 1985; Rouviere and Chen, 2003). Cyclohexane has the most stable chemical structure of all cycloalkanes, due to the lowest energy strain of the C–C-bonds (Bruice, 2004). Cyclohexane has a low solubility in water (0.68 mM at 25°C), and is a relatively volatile hydrocarbon (boiling point 80.7°C) (Dean, 1992). A relatively low number of aerobic bacterial strains able to degrade cyclohexane have been isolated. These were affiliated with the Actinobacteria (Stirling et al., 1977), or with the Proteobacteria (Anderson et al., 1980; Trower et al., 1985; Rouviere and Chen, 2003). Under aerobic conditions, cylohexane is activated by a cyclohexane monooxygenase forming cyclohexanol, which is further oxidized to cyclohexanone, caprolactone and adipate (Perry, 1984; Cheng et al., 2002). Recent studies indicated that aerobic cyclohexane-degrading bacteria may be among the first microorganisms to be enriched *in situ* as a response to oil spills. During the early stages of the Deepwater Horizon oil spill, hydrocarbons released into deep-sea waters led to the *in situ* enrichment of an uncultivated group of *Oceanospirillales* (Hazen et al., 2010; Redmond and Valentine, 2012). Subsequent single-cell genome sequencing showed that the enriched *Oceanospirillales* contained a near-complete pathway for cyclohexane oxidation (Mason et al., 2012). In addition, genes coding for the cyclohexane degradation pathway were found in metagenome and metatranscriptome libraries, indicating that cyclohexane-degrading bacteria were abundant and active in the crude oil contaminated waters (Mason et al., 2012).

Anaerobic degradation of cycloalkanes was shown with a sulfate-reducing enrichment culture obtained with ethylcyclopentane from a gas condensate-contaminated aquifer (Rios-Hernandez et al., 2003), and with a nitrate-reducing enrichment culture growing with cyclohexane, obtained from freshwater sediments (Musat et al., 2010). In addition, anaerobic degradation of cycloalkanes was demonstrated using sediment samples from a hydrocarbon-contaminated aquifer provided with gasoline and gas condensates (Townsend et al., 2004). Under anaerobic conditions, cycloalkanes are activated by addition to fumarate yielding cycloalkylsuccinate derivatives, a mechanism similar to that of *n*-alkane activation catalyzed by glycyl radical enzymes (Rabus et al., 2001; Callaghan et al., 2008; Grundmann et al., 2008; Jarling et al., 2012). This was demonstrated by metabolite analyses of a sulfate-reducing enrichment culture growing with ethylcyclopentane (Rios-Hernandez et al., 2003), and of a nitrate-reducing enrichment culture with cyclohexane (Musat et al., 2010). In addition, co-activation of cyclopentane to cyclopentylsuccinate was observed in cultures of a denitrifying strain growing with *n*-alkanes from crude oil, while cyclopentane alone did not serve as a growth substrate (Wilkes et al., 2003).

Anaerobic pure cultures able to grow with cycloalkanes have not been reported so far. Phylotypes related to *Desulfotomaculum* sp., identified by DGGE analyses, have been proposed to be involved in ethylcyclopentane degradation in a sulfate-reducing enrichment culture (Rios-Hernandez et al., 2003). Based on hybridization with oligonucleotide probes, a *Geobacter* sp.-related phylotype was proposed to be responsible for cyclohexane degradation in a nitrate-reducing enrichment culture (Musat et al., 2010). Nevertheless, the identity of anaerobic cycloalkane-degrading microorganisms is largely unknown. Such microorganisms have not been identified so far in marine environments, despite the fact that the oceans receive massive inputs of crude oil (for example the recent Deepwater Horizon spill, e.g., Atlas and Hazen, 2011). In the present study, we enriched cyclohexane-degrading, sulfate-reducing bacteria from marine sediments contaminated with hydrocarbons. The microorganisms in the enrichment culture were identified by hybridization with sequence-specific oligonucleotide probes. We analyzed the ability of the enriched bacteria to degrade other cyclic and *n*-alkanes, by short-term incubations with dense-cell suspensions. Also, we investigated the pathway of cyclohexane activation and further degradation, by analysis of metabolites. Based on the metabolites detected, we propose a pathway for cyclohexane degradation under sulfate-reducing conditions.

## Materials and methods

### Chemicals

Cyclopentane, cyclohexane, methylcyclopentane, methylcyclohexane, *n*-pentane, *n*-hexane, benzene and toluene of analytical grade were purchased from Merck (Damstadt, Germany). 2,2,4,4,6,8,8-Heptamethylnonane (HMN), cyclohexylsuccinic acid, 3-cyclohexylpropionic acid and cyclohexanecarboxylic acid were purchased from Sigma-Aldrich (Steinheim-Germany). The gaseous alkanes ethane, propane and *n*-butane of purity 3.5 were purchased from Air Liquide (Düsseldorf, Germany).

### Source of organisms, culture media and cultivation techniques

Anoxic sediment from a hydrocarbon-contaminated lagoon in the Mediterranean was used to establish enrichment cultures. Collected sediment was homogenized by mixing with a metal spoon in an anoxic tent, under a N_2_:CO_2_ (9:1, v/v) atmosphere. Enrichment cultures were established in 100 ml flat glass bottles containing 50 ml of defined NaHCO_3_/CO_2_-buffered artificial sea water medium (Widdel and Bak, 1992), 5 ml homogenized sediment as inoculum, and a headspace of N_2_:CO_2_ (9:1, v/v). The bottles were sealed with butyl-rubber stoppers, and provided with 2.5 ml of HMN as an inert carrier phase, containing 0.5% (v/v) cyclohexane. Control bottles without addition of cyclohexane were set up in a similar way. Bottles were incubated at 28°C in a nearly horizontal position, in order to avoid direct contact of the carrier phase with the stoppers, and to maximize the surface of the carrier-medium interface (Rabus and Widdel, 1995). Subsequent cultures were inoculated with 10% (v/v) of an active culture. Cultures containing sediment were incubated without shaking, while later sediment-free cultures were incubated with slow (60 rpm) horizontal shaking. Sediment-free cultures were amended with 3 ml l^−1^ trace element solution (Widdel and Bak, 1992). Quantitative growth experiments were set up in 120-ml round flat bottles containing 90 ml medium, 10 ml inoculum and 5 ml HMN with 0.5 or 0.2% (v/v) cyclohexane. Sterile bottles with cyclohexane and inoculated bottles without addition of cyclohexane were used as controls. For extraction of metabolites, cultures were prepared in a similar manner as the quantitative growth experiments, with the use of culture medium with a limited amount of sulfate (5 mM).

Substrate tests with other hydrocarbons were performed with 15× concentrated cell suspensions. For preparation of concentrated cell suspensions, a total volume of 2100 ml of cyclohexane-grown cultures was separated inside an anoxic tent (N_2_:CO_2_ 9:1 v/v) from the carrier phase using a separatory funnel, centrifuged, and suspended in 140 ml of anoxic medium. Aliquots of 10 ml were distributed in 15 ml butyl stoppered cultivation tubes and provided with the following substrates (concentration given as percent v/v in HMN, unless otherwise stated): cyclopentane (0.2%), methylcyclopentane (0.5%), methylcyclohexane (0.5%), ethane (5 ml gas in the headspace), propane (5 ml gas in the headspace), *n*-butane (5 ml gas in the headspace), *n*-pentane (1%), *n*-hexane (1%), benzene (0.5%), and toluene (0.5%). For the substrates that were added dissolved in HMN, a volume of 0.5 ml HMN containing the indicated substrate concentration was added per tube. Unamended controls were prepared in a similar manner. In order to provide the cells with a starting substrate, assuming that enzymes other than those involved in cyclohexane degradation might be required for the degradation of the tested substrates, all cultures, including the negative controls were amended with 50 μl of HMN with 0.5% (v/v) cyclohexane (10% of the regular amount of cyclohexane), as a starter substrate. Substrate tests results were confirmed with two independent incubations.

### Analytical methods

Sulfide concentrations were determined by photometric measurements (λ = 480 nm) of colloidal CuS, as described elsewhere (Cord-Ruwisch, 1985). Cyclohexane concentrations in HMN were measured by headspace analysis. The measurement is based on gas phase equilibrium with the carrier phase at constant temperatures (28°C). Volumes of 0.1 ml headspace were withdrawn with gas-tight, N_2_-flushed syringes, and injected without a split into a Shimadzu GC-14B gas chromatograph, equipped with a Supel-Q PLOT column (30 m × 0.53 mm, 30 μm film thickness; Supelco, Bellefonte, USA) and a flame ionization detector. The oven temperature was maintained at 140°C, and the injection and detection temperatures were maintained at 150 and 280°C, respectively. The carrier phase was N_2_ at a flow rate of 3 ml min^−1^. Samples were analyzed in triplicates. An external calibration was built by gas phase measurements of bottles with defined concentrations of cyclohexane in HMN, equilibrated at 28°C.

Prior to extraction of metabolites, cultures were inactivated by heating on a water bath at 85°C for 30 min, cooled down to room temperature and acidified using HCl to a pH of about 2. The culture medium was separated from the HMN phase with separatory funnels, and extracted three times with 1.5 volumes of dichloromethane (Rabus et al., 2001; Wilkes et al., 2002). The extracts were combined and dried over anhydrous Na_2_SO_4_. Sterile cultures with cyclohexane and inoculated cultures without cyclohexane were used as controls. Extracts were methylated using a solution of diazomethane in diethyl ether and subsequently analyzed using a Trace GC-MS system (Thermo Scientific, Bremen, Germany). The gas chromatograph was equipped with a 5% phenyl polysilphenylene-siloxane capillary column (BPX-5, SGE; 50 m × 0.22 mm, 0.25 μm film thickness). The column temperature was initially held at 50°C for 1 min, then heated to 310°C at a rate of 3°C min^−1^ with a final hold time of 30 min. Helium was used as the carrier gas. The PTV injector temperature was programmed from 50 to 300°C (10 min isothermal) at a rate of 10°C s^−1^, and the injection volume was 1 μl in splitless mode. Mass spectra were recorded from *m/z* 50 to 600. Identification of metabolites (as methyl esters) was based on comparison of retention times and mass spectra with those of authentic standards.

### Construction of 16S rRNA gene libraries and phylogenetic analysis

Genomic DNA was extracted from the enrichment culture and used in PCR reactions to amplify nearly full-length 16SrRNA genes with bacteria-specific primers (Musat et al., 2006). The PCR products were purified using the QIAquick Gel Extraction kit (Qiagen, Hilden, Germany), cloned into the pCR4 vector (TOPO-TA cloning kit, Invitrogen, Groningen, Netherlands) and transformed into *E. coli* Top 10 competent cells (Invitrogen). Positive clones were sequenced using the ABI Prism BigDye Terminator v 3.0 cycle sequencing kit and an ABI Prism 3100 Genetic Analyzer (Applied Biosystems, Darmstadt, Germany). Gene libraries were screened for phylotypes affiliated with the Deltaproteobacteria by partial sequencing with the primer 517f (Muyzer et al., 1993). Clones affiliated with the Deltaproteobacteria were fully sequenced using the primers M13F and M13R (Invitrogen). Sequences were assembled with the DNA Baser software (www.dnabaser.com) and analyzed using the BLAST(N) algorithm (Altschul et al., 1990). For phylogenetic reconstruction, nearly full-length sequences (>1300 bp) were aligned to those of the Silva database (Pruesse et al., 2007). Phylogenetic trees were constructed in ARB (Ludwig et al., 2004) using neighbor joining, maximum likelihood and maximum parsimony, and by applying different sets of filters. The sequence data have been deposited in the DDBJ, EMBL and GenBank databases under accession numbers KP009598–KP009615.

### Microscopy and whole cell hybridization

For phase-contrast images 10 μl aliquots from living cultures were transferred onto agar-coated (1% w/v) glass slides and covered with a cover slip. Cells were examined with a Zeiss Axioskop 50 microscope (Zeiss; Oberkochen, Germany). For whole cell hybridization, cells were fixed with 2% paraformaldehyde in 1× phosphate-buffered saline (PBS; 10 mM sodium phosphate pH 7.2, 130 mM NaCl) for 1 h at room temperature, washed with 1× PBS, and stored in 1× PBS–ethanol (1:1) at –20°C. Aliquots of fixed cells were filtered onto 0.2 μm pore GTTP polycarbonate filters (Millipore, Eschborn-Germany). Cells on filters were hybridized with Horseradish peroxidase (HRP)-labeled 16S rRNA-targeted oligonucleotide probes. Signal amplification was done as described elsewhere (Pernthaler et al., 2002) using Alexa 488® tyramides (Invitrogen). Thereafter, all cells were additionally stained with DAPI and microscopically counted as previously described (Snaidr et al., 1997). The hybridized, DAPI-stained cells were analyzed with a Zeiss Axioskop 2 mot plus fluorescence microscope (Zeiss), using a HC F36-525 filter (AHF, Tübingen, Germany) for probe signal recording and a F81-360 filter (AHF, Tübingen, Germany) for DAPI signal recording. Images were acquired and processed using the Zeiss Axio Vision 4.0 software release 4.6.3 (Carl Zeiss Imaging Solutions; Hallbergmoos, Germany). The oligonucleotide probes used in this study, Cyhx28-EdB_152 (AGCAAGCCTTTCAGCATG; sequence-specific, this study), DSS658 (Manz et al., 1998), EUB338 (Amann et al., 1990) and NON338 (Amann et al., 1990) were purchased from Biomers GmbH (Ulm, Germany). The sequence-specific probe designed in this study (in ARB, after Hugenholtz et al., 2001), was evaluated for specificity in hybridization assays with increasing formamide [FA] concentrations (0-60%, with 10% increment) with the enrichment culture as a positive target. The highest FA concentration where strong fluorescent probe signals were still observed was 20% FA. Cultured strains with one or two mismatches were not available to serve as negative controls.

## Results and discussions

### Enrichment and phylogenetic analyses of cyclohexane-degrading sulfate-reducing bacteria

Enrichment of cyclohexane-degrading bacteria with sulfate as the terminal electron acceptor was started with intertidal, hydrocarbon-contaminated marine sediments. Sulfide concentrations were monitored as a measure of cyclohexane-dependent sulfate reduction. After approximately 5 months of incubation, the incubation with added cyclohexane formed ca. 15 mM sulfide vs. 8 mM in the control incubation without cyclohexane. A sediment-free enrichment culture (Cyhx28-EdB), obtained by repeated transfers in fresh culture medium, formed 15 mM sulfate within an incubation time of approximately 8 weeks (data not shown).

**Figure 1:**
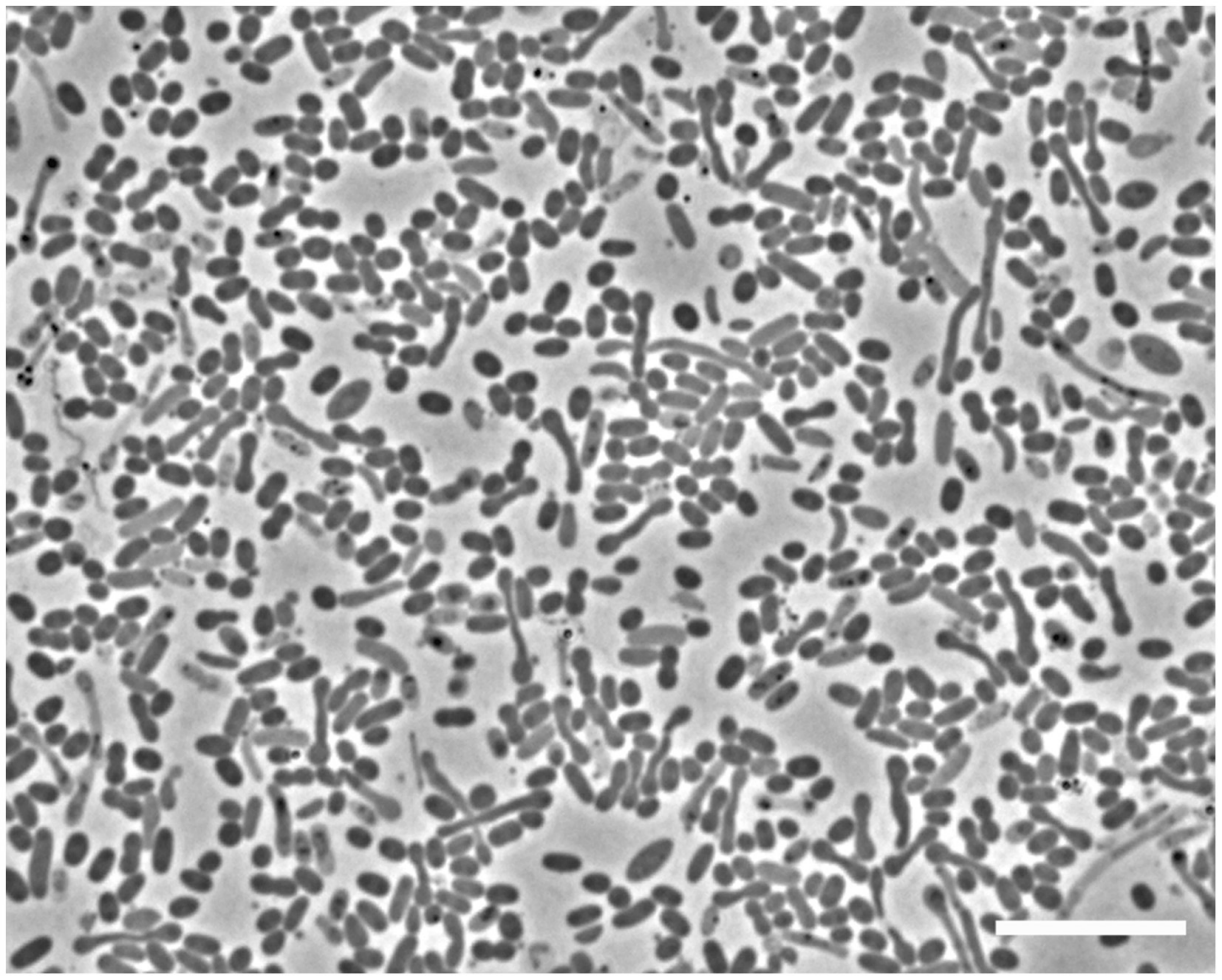
**Phase contrast microscopic image of cells in the enrichment culture Cyhx28-EdB**. The enrichment appeared dominated by oval cells (1.5 × 0.8 μm, on average). Upon depletion of cyclohexane and accumulation of high concentrations of sulfide, elongated cells could be observed. Scale bar = 10 μm.

**Figure 2:**
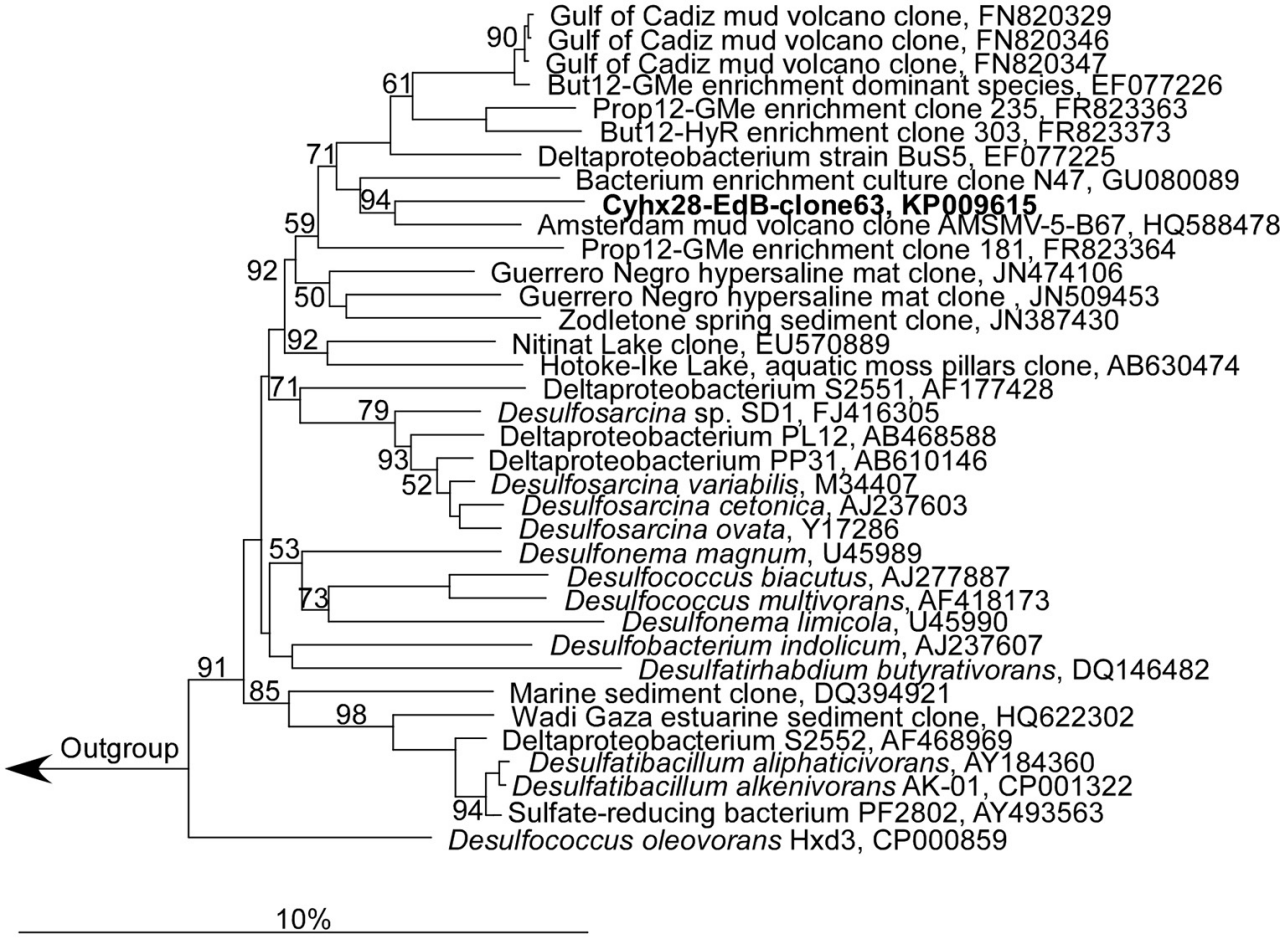
**Phylogenetic affiliation of the most abundant phylotype in the enrichment culture, Cyhx28-EdB-clone63 (marked in bold-face)**. The phylogenetic tree was calculated in ARB by neighbor-joining, using only nearly full-length sequences (>1300 nt), with application of different sets of filters. The numbers next to nodes indicate bootstrap values higher than 50%. The scale bar represents 10% estimated sequence divergence.

Microscopic analyses showed an apparent dominance by oval to rod-shaped cells of 1.5 μm length × 0.8 μm diameter, on average (Figure 1). Upon depletion of cyclohexane and formation of high concentrations of sulfide some cells showed an elongated morphology (Figure 1). Since attempts to isolate the cyclohexane-degrading microorganism in pure culture were not successful, the enrichment culture was further analyzed by molecular biology methods. Construction and analysis of a 16S rRNA gene library (*n* = 96) showed that most of the sequences (*n* = 53) were affiliated with the Deltaproteobacteria. Of the Deltaproteobacterial sequences, the majority (*n* = 42) were affiliated with the *Desulfosarcina-Desulfococcus* clade (>94% sequence identity). Other Deltaproteobacteria-affiliated sequences were closely related to *Desulfobacterium anilini* (*n* = 4) and *Desulfotignum balticum* (*n* = 6) (Figure SI1). A large number of clones (*n* = 37) were closely related to sequences belonging to the OP3 cluster, a group within the *Planctomycetes/Verrucomicrobia/Chlamydiae* superphylum, from which no isolated strains have been reported so far (e.g., Rotaru et al., 2012). In an attempt to identify the putative most abundant microorganisms, the 16S rRNA gene was amplified using increasingly diluted DNA template. The PCR products from the highest template dilution yielding a result (10^−2^) were sequenced without cloning. The sequences obtained were identical with one of the clones affiliated with the *Desulfosarcina-Desulfococcus* cluster, Cyhx28-EdB-clone63 (Figure 2). Cyhx28-EdB-clone63 was closely related to sequences from the Amsterdam mud volcano (94.7% identity; (Pachiadaki et al., 2011), Gulf of Cadiz mud volcano (>93% identity), clone sequences from a naphthalene-degrading enrichment culture (Selesi et al., 2010), Guerrero Negro hypersaline mat clones (Harris et al., 2013) and Zodletone spring sediment clones (Youssef et al., 2012). The closest cultivated relatives were strain BuS5 (92.7% identity; Kniemeyer et al., 2007), and dominant phylotypes in propane- and *n*-butane-degrading enrichment cultures from the Gulf of Mexico and Hydrate Ridge sediments (Kniemeyer et al., 2007; Jaekel et al., 2013). Substrate tests with strain BuS5 showed that this microorganism degraded only the gaseous alkanes propane and *n*-butane (Kniemeyer et al., 2007). Shorter (ethane and methane) or longer (*n*-pentane and higher) alkanes did not serve as growth subtstrates. A similarly restricted substrate range was found for the enrichment cultures Prop12-GMe, But12-GMe, and But12-HyR, which also degraded only propane and *n*-butane (Jaekel et al., 2013).

**Figure 3:**
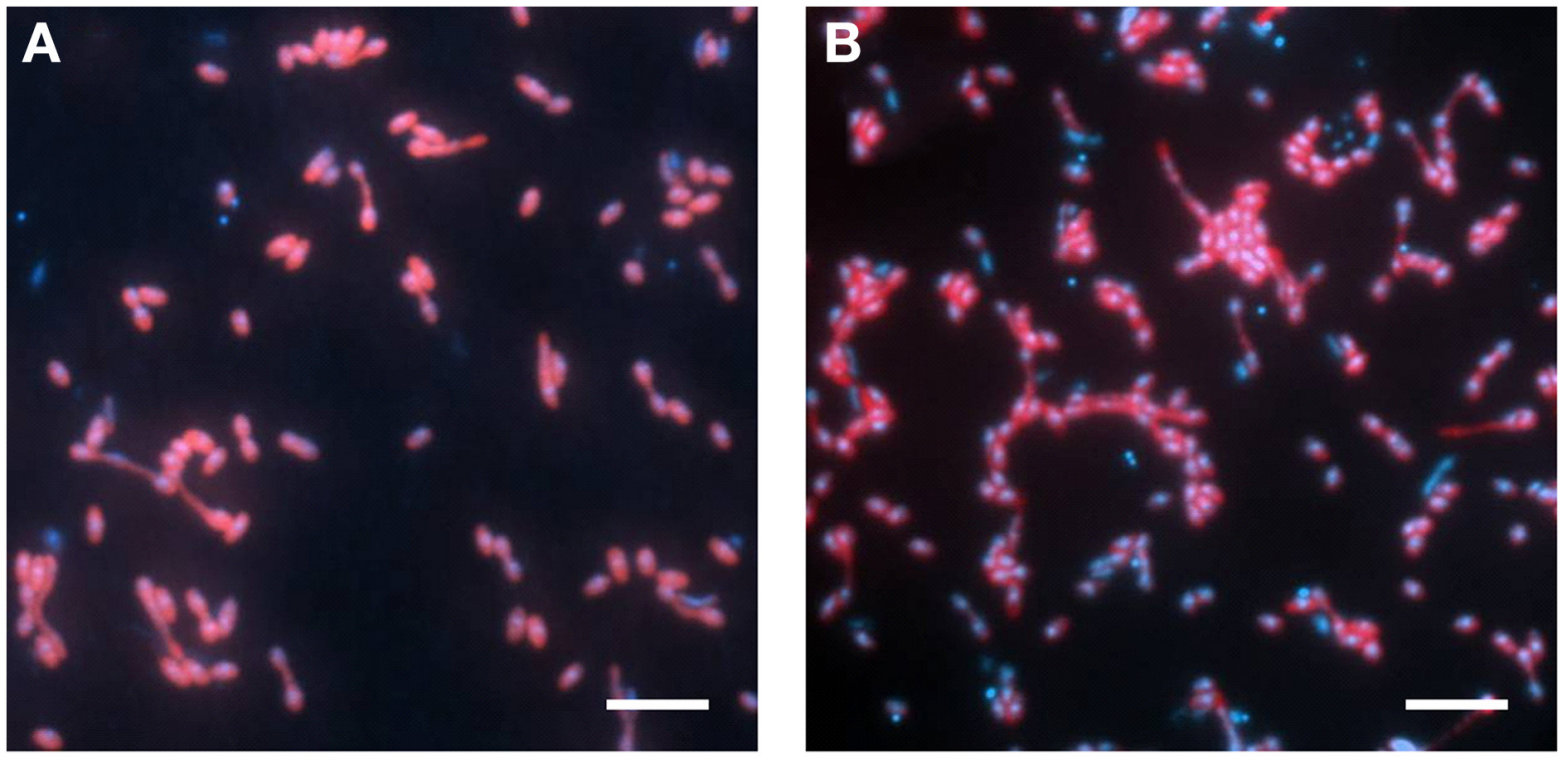
**Whole-cell hybridization (CARD-FISH) with the group-specific probe DSS658 (A) and with the sequence-specific probe Cyhx28-EdB_152 (B), showing the dominance of the phylotype Cyhx28-EdB-clone63**. The images show an overlay of probe (red) and DAPI (blue) signals. Scale bars = 5 μm.

The abundance of the phylotype represented by Cyhx28-EdB-clone63 in the enrichment culture was further quantified by whole-cell hybridization. Hybridizations with the group-specific oligonucleotide probe DSS658, targeting most of the bacteria afiliated with the *Desulfosarcina-Desulfococcus* cluster, including Cyhx28-EdB-clone63, showed that this phylogenetic group accounted for 84.3% of the total cell number determined by DAPI staining (Figure 3). Further hybridizations with the sequence-specific oligonucleotide probe Cyhx28-EdB_152 showed that the phylotype Cyhx28-EdB-clone63 accounted for 80.2% of the total cell number (Figure 3). High abundance of single phylotypes has been found in other anaerobic, hydrocarbon-degrading enrichment cultures, for example with benzene under sulfate-reducing conditions (Musat and Widdel, 2008), or with alkylbenzenes and *n*-alkanes under denitrifying conditions (Rabus et al., 1999). In addition, highly abundant phylotypes in enrichment cultures of sulfate-reducing bacteria degrading gaseous alkanes have been shown to be directly involved in hydrocarbon degradation by incubations with ^13^C-labeled substrates followed by nanoSIMS analyses (Jaekel et al., 2013). Considering the high abundance of the phylotype Cyhx28-EdB-clone63, we propose that it plays a very important role in the biodegradation of cyclohexane.

### Stoichiometry of cyclohexane degradation

**Figure 4:**
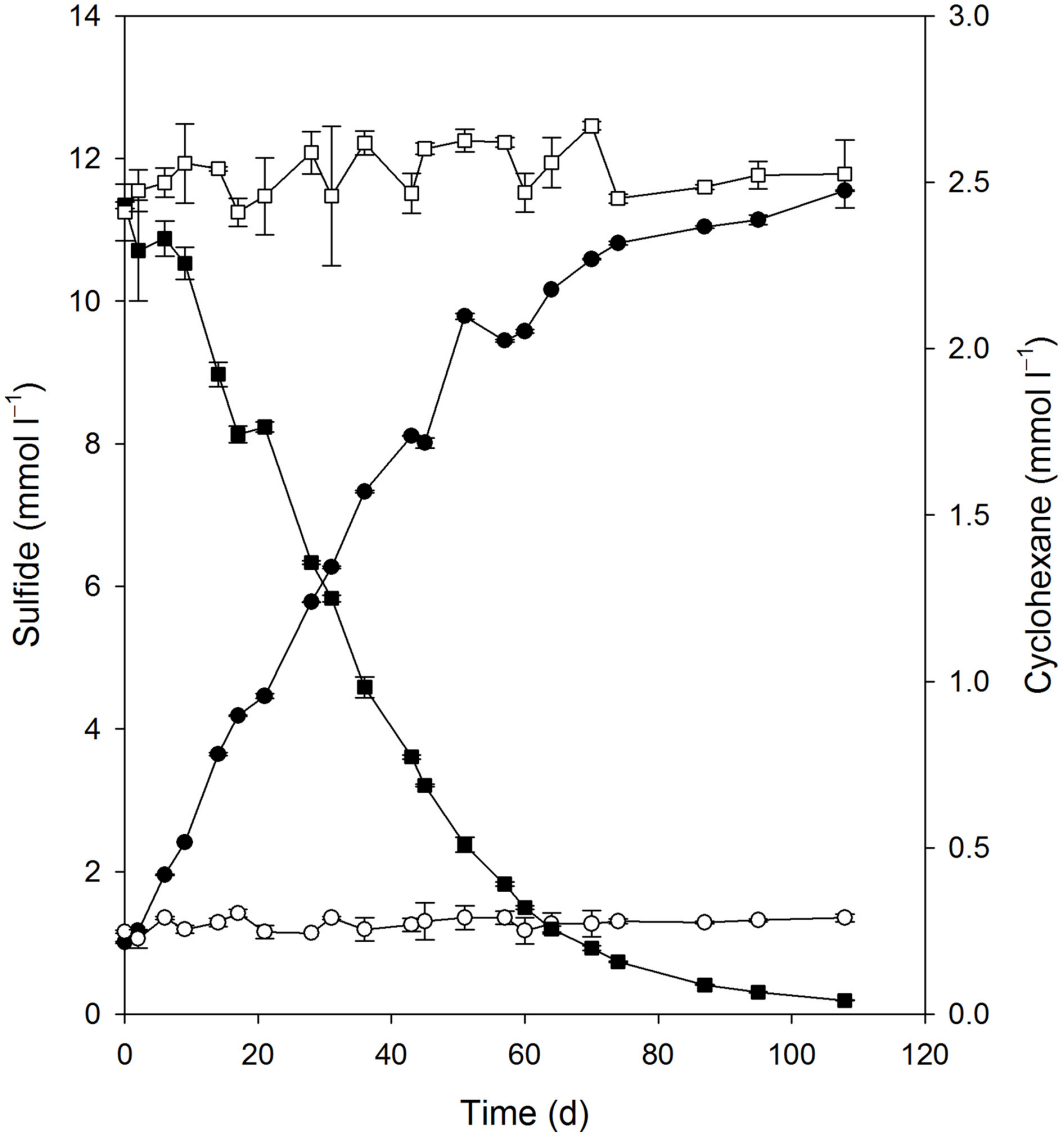
**Consumption of cyclohexane (■) and reduction of sulfate to sulfide (•) in the enrichment culture Cyhx28-EdB**. The cultures were set up in anoxic bottles, with a culture volume of 100 ml, and 5 ml heptamethylnonane as a carrier phase containing 0.5% v/v cyclohexane. Cyclohexane was not lost in an abiotic control (□), and inoculated cultures without cyclohexane did not show sulfide production (○). The data represent averages of triplicate cyclohexane and sulfide measurements; vertical bars indicate standard deviations.

Quantitative growth experiments showed depletion of cyclohexane coupled to the reduction of sulfate to sulfide within about 100 days (Figure 4). No cyclohexane was consumed in sterile controls, and minor amounts of sulfide were produced in inoculated control cultures without addition of cyclohexane, probably due to carry over of small amounts of substrate during inoculation (Figure 4, Table 1). Calculation of the net electron balance from incubations with different cyclohexane concentrations (2.4 and 1.0 mmol l^−1^) yielded ratios close to the theoretical one for complete oxidation of cyclohexane to CO_2_ according to eq. 1 (Table 1).

(1) $$\begin{array}{l} 2C_{6}H_{12}+9SO_{4}^{2-}\to 12HCO_{3}^{-}+9HS^{-}+3H^{+}\\ \;\Delta G^{{{}^{\circ}}^{\prime }}=-202.6kJ\;{\left(\text{mol cyclohexane}\right)}^{-1} \end{array}$$

**Table 1:** **Electron balance of the observed degradation of cyclohexane coupled to the reduction of sulfate to sulfide**.

**Cyclohexane, Sulfate, Electrons (mmol l^−^1)**	**Cyhx28-EdB + 0.5% C_6_H_12_ (v/v in HMN)**	**Cyhx28-EdB + 0.2% C_6_H_12_ (v/v in HMN)**	**Cyhx28-EdB – C_6_H_12_**	**Abiotic control + 0.5% C_6_H_12_ (v/v in HMN)**
Supplied C_6_H_12_	2.4	1.0	–	2.4
Consumed C_6_H_12_	2.4	1.0	–	0
Electrons from C_6_H^a^_12_	86.4	36.0	–	–
Supplied SO^2−^_4_	28.0	28.0	28.0	–
Consumed SO^2−^^b^_4_	10.5	4.7	0.2	–
Electrons for SO^2−^^c^_4_	82.4	36.0	–	–
Electron balance^d^	0.95	1.00	–	–

*Parallel experiments were carried out, with different starting amounts of cyclohexane*.

a *Electrons from consumed C_6_H_12_ were calculated considering the complete oxidation: C_6_H_12_ +12 H_2_O → 6CO_2_ + 36H^+^ + 36e^−^*.

b *The amount of consumed SO^2−^_4_ was determined by quantification of produced H_2_S (corrected for the H_2_S added as a reducing agent in the culture medium, 1 mmol l^−1^)*.

c Electrons for SO^2−^_4_ reduction were calculated considering: SO^2−^_4_ + 8e^−^ + 9H^+^ → HS^−^ + 4H_2_O. For calculation, the H_2_S produced in cultures with added cyclohexane was corrected for the H_2_S produced in C_6_H_12_-free bottles

d *Electrons consumed by SO^2−^_4_ reduction divided by electrons from C_6_H_12_ consumed*.

The observed stoichiometry could be due to complete oxidation of cyclohexane by the proposed microorganism, Cyhx28-EdB-clone63, or to incomplete cyclohexane oxidation followed by the scavenging of the intermediates by other sulfate-reducing bacteria in the enrichment culture. The sulfate-reducing bacteria isolated so far with aliphatic or aromatic hydrocarbons as substrates are complete oxidizers, degrading the hydrocarbon substrate completely to CO_2_ (for an overview see Widdel et al., 2010). Among these are strains affiliated to the same phylogenetic group as the phylotype Cyhx28-EdB-clone63 (*Desulfosarcina-Desulfococcus*), degrading gaseous alkanes (strain BuS5 and dominant phylotypes in propane- and n-butane-degrading enrichment cultures; (Kniemeyer et al., 2007; Jaekel et al., 2013), *n*-alkanes >C_6_ (e.g., Aeckersberg et al., 1991; So and Young, 1999), or aromatic hydrocarbons (e.g., Harms et al., 1999). Complete oxidation of cycloalkanes under sulfate-reducing conditions has been reported so far with an enrichment culture growing with ethylcyclopentane (Rios-Hernandez et al., 2003). Based on these studies, the present calculations of the net electron balances for different cyclohexane concentrations, and the high abundance of the phylotype Cyhx28-EdB-clone63, we propose that the dominant phylotype has the ability to degrade cyclohexane completely to CO_2_; the other microorganisms in the enrichment culture may grow at the expense of excreted metabolites or dead biomass of the dominant phylotype. For example, recent metagenomic analyses of the OP3, which are the second most abundant phylogenetic group in the present enrichment culture based on clone frequency, identified genes typical of the tricarboxylic acid cycle (Glockner et al., 2010). One may speculate that the OP3 scavenge low-molecular mass fatty acids from the downstream cyclohexane degradation pathway.

### Substrate tests with hydrocarbons other than cyclohexane

**Figure 5:**
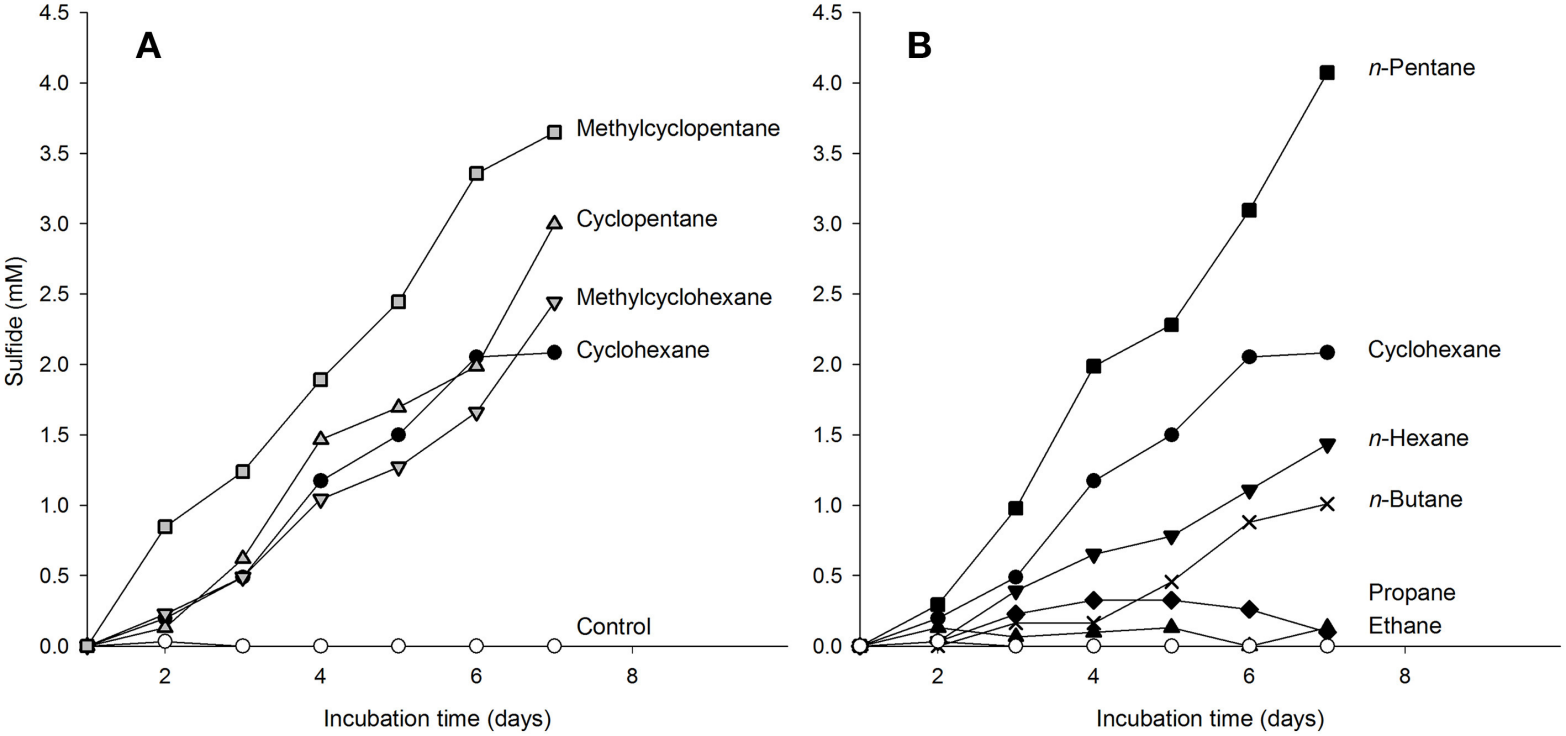
**Response of cyclohexane-grown, dense-cell suspensions of the enrichment culture Cyhx28-EdB to additions of cycloalkanes (A), and *n*-alkanes (B), determined as hydrocarbon-dependent sulfate reduction**. Cyclohexane-dependent sulfate reduction is shown in both panels as reference (•). The enrichment culture was apparently able to use all cyclic alkanes tested **(A)**, as well as *n*-pentane and *n*-hexane **(B)**. A response to addition of *n*-butane was recorded after a lag phase of 4 days **(B**, ×). No sulfide production could be detected in incubations with ethane, propane **(B)**, and in substrate-free controls (◯, in both panels). The experiments were performed in anoxic tubes with 10 ml of a 15 × concentrated cell suspension, and confirmed by two independent incubations (here only one data set is shown). CARD-FISH with the sequence-specific probe Cyhx28-EdB_152 showed that the phylotype Cyhx28-EdB-clone63 was dominant in all positive test cultures at the end of the incubation time (Figure SI2).

We tested the ability of the enrichment culture Cyhx28-EdB to degrade other hydrocarbons than cyclohexane. To prevent false positive results by the enrichment of microorganisms other than the dominant phylotype upon addition of new substrates, the experiments were done with concentrated cell suspensions and incubated for a relatively short time. The enrichment culture responded without a lag phase to additions of other cyclic alkanes, e.g., cyclopentane, methylcyclopentane and methylcyclohexane (Figure 5A). Of the *n*-alkanes tested, the enrichment culture was apparently able to grow with *n*-pentane and *n*-hexane (Figure 5B). The enrichment culture showed *n*-butane-dependent sulfate-reduction after a lag phase of 4 days (Figure 5B). No sulfate reduction could be detected in incubations with ethane or propane (Figure 5B), or with the aromatic hydrocarbons benzene and toluene (not shown). Hybridizations with the specific probe Cyhx28-EdB_152 showed that the phylotype Cyhx28-EdB-clone63 was highly abundant in all positive substrate test incubations (Figure SI2). These results suggest that the phylotype Cyhx28-EdB-clone63 was most likely responsible for the degradation of the tested hydrocarbons. To date, all reports about microorgansims capable of degrading cycloalkanes under anaerobic conditions showed degradation of single substrates, such as cyclohexane (Musat et al., 2010), ethylcyclopentane (Rios-Hernandez et al., 2003), or cyclopentene, methylcyclopentene, methylcyclopentane, cyclohexane and methylcyclohexane by distinct sulfate-reducing enrichment cultures (Townsend et al., 2004). In addition, it has been reported that the nitrate reducing strain HxN1 is able to co-activate (but not grow with) cyclopentane and methylcyclopentane during growth on *n*-hexane or crude oil (Wilkes et al., 2003). Given these results and the dominance of the phylotype Cyhx28-EdB-clone63 (>80%), we hypothesize that Cyhx28-EdB-clone63 is relatively versatile with respect to the range of hydrocarbons utilized, including C_4_-C_6_*n*-alkanes as well as methyl substituted and unsubstituted five- and six-ring cycloalkanes. Future studies should establish whether other cycloalkane degraders also display a broad substrate range as proposed for the phylotype Cyhx28-EdB-clone63.

The slower sulfate reduction with *n*-hexane vs. cyclohexane may have been caused by the lower solubility in water of the former (*n*-hexane 0.14 mmol l^−1^, cyclohexane 0.68 mmol l^−1^, at 25°C; data from Eastcott et al., 1988). However, solubility alone cannot explain the observed differences in sulfate reduction with the different substrates. For example, of the hydrocarbons tested cyclopentane has the highest solubility (2.28 mmol l^−1^, McAuliffe, 1966; Eastcott et al., 1988), but the sulfate reduction profile with cyclopentane was very similar to that of cyclohexane (Figure 5A). Also, the highest sulfate reduction rate was measured with *n*-pentane, which has a solubility in water similar to that of cyclohexane (0.56 mmol l^−1^, Eastcott et al., 1988). We rather explain the differences in the sulfate reduction rates as an effect of substrate affinity of the activating enzyme, as shown before in enzyme assays with the (1-methyl)alkylsuccinate synthase from strain HxN1 (Webner, 2012). The more pronounced delayed response to *n*-butane (solubility in water at 25°C, 1.22 mmol l^−1^) may also be due to differences in substrate affinity. However, sulfate reducing bacteria able to utilize *n*-alkanes ≥ C_6_ isolated so far are not able to grow with the short-chain, gaseous alkanes propane and *n*-butane (Widdel et al., 2010). Also, sulfate reducers able to degrade propane and *n*-butane appear to be restricted to these hydrocarbons (Kniemeyer et al., 2007; Jaekel et al., 2013). We may therefore speculate that the delayed response to *n*-butane could have been caused by the induction of different activating enzymes. The presence of two operons encoding for methylalkylsuccinate synthases was so far demonstrated by genome analyses of the alkane-degrading sulfate-reducing bacterium *D. alkenivorans* strain AK-01 (Callaghan et al., 2012). The encoded enzymes may display different affinities to various hydrocarbon substrates, and may not be constitutively expressed.

### Analyses of metabolites—evidence for the activation mechanism of cyclohexane under anaerobic conditions

GC-MS analysis of derivatized extracts from cultures grown with cyclohexane in sulfate-limited medium showed the presence of organic acids which were absent in controls without added cyclohexane and in sterile controls. We detected a metabolite whose mass spectrum showed significant fragment ions at *m/z* 114, 146, 155, and 197 being in agreement with the structure of cyclohexylsuccinic acid (detected as cyclohexylsuccinic acid dimethyl ester, Figure SI3 and Musat et al., 2010) which was subsequently confirmed by comparison with a standard. The finding of cyclohexylsuccinic acid indicates that the Cyhx28-EdB-clone63-dominating culture activates cyclohexane by addition to fumarate. This mechanism of activation is thus similar to the activation of linear saturated hydrocarbons by addition to fumarate at the secondary (subterminal) carbon atom, demonstrated for the first time with *n*-alkane-degrading cultures of nitrate- (Rabus et al., 2001) and sulfate-reducing bacteria (Kropp et al., 2000). Activation by addition to fumarate, which so far appears as the most widespread mode of activation of alkanes (Widdel and Grundmann, 2010), was also demonstrated for the degradation of propane and *n*-butane by strain BuS5, the closest cultured relative of the Cyhx28-EdB-clone63 (Kniemeyer et al., 2007). Activation of cycloalkanes by addition to fumarate was previously reported with a sulfate-reducing enrichment culture degrading ethylcyclopentane (Rios-Hernandez et al., 2003). Cyclohexane activation by the same mechanism was for the first time reported with a nitrate-reducing enrichment culture dominated by *Geobacter* spp. and Anammox microorganisms (Musat et al., 2010). In addition, methylcyclopentane and cyclopentane, although not serving as growth substrates, were co-activated by addition to fumarate by the nitrate-reducing strain HxN1 during growth on *n*-hexane or crude oil (Wilkes et al., 2003).

**Figure 6:**
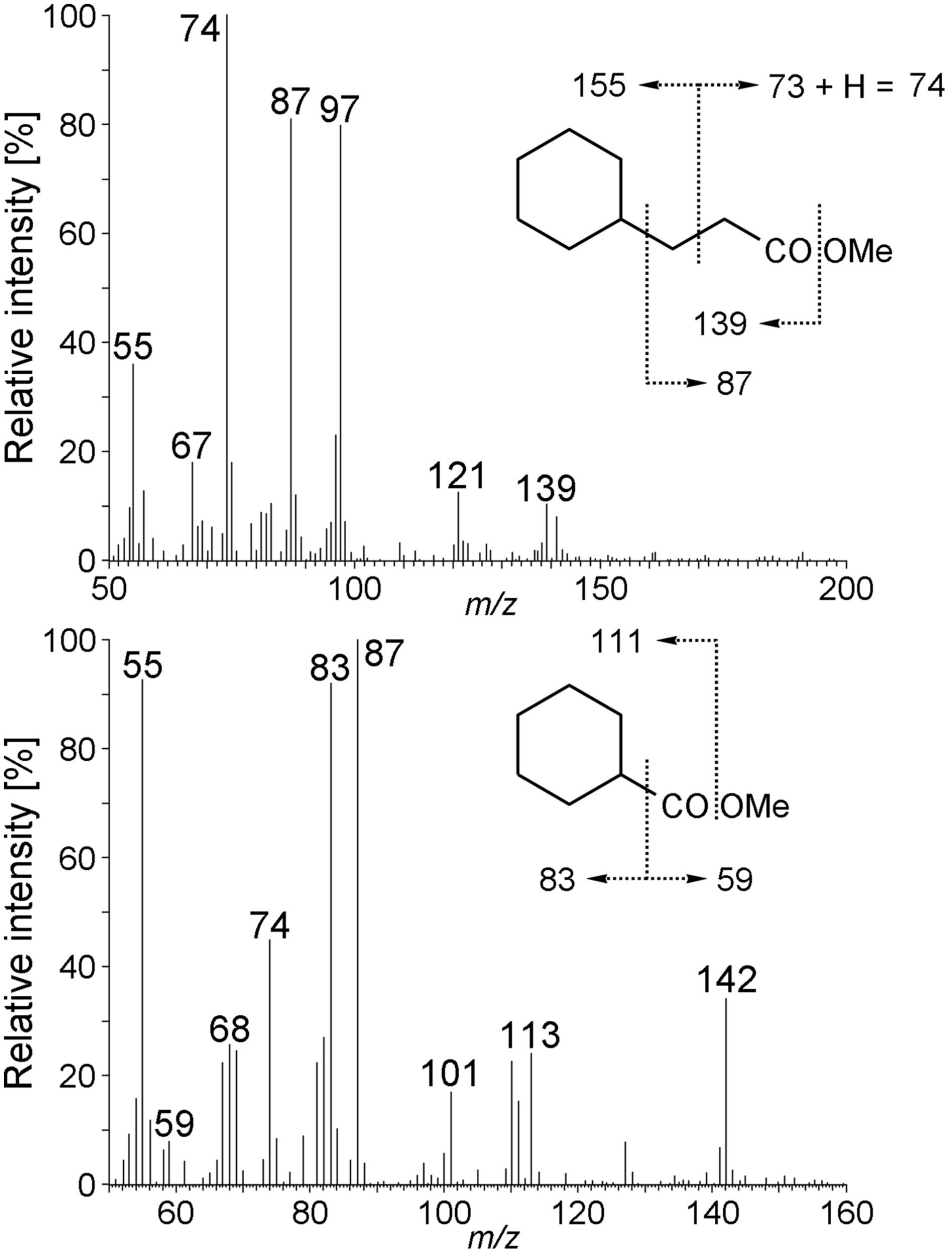
**Mass spectra of 3-cyclohexylpropionic acid methyl ester, and cyclohexanecarboxylic acid methyl ester derived from the Cyhx28-EdB enrichment culture grown with cyclohexane**. The mass spectra and GC retention times of these metabolites were identical to those of standard compounds (data not shown).

**Figure 7:**
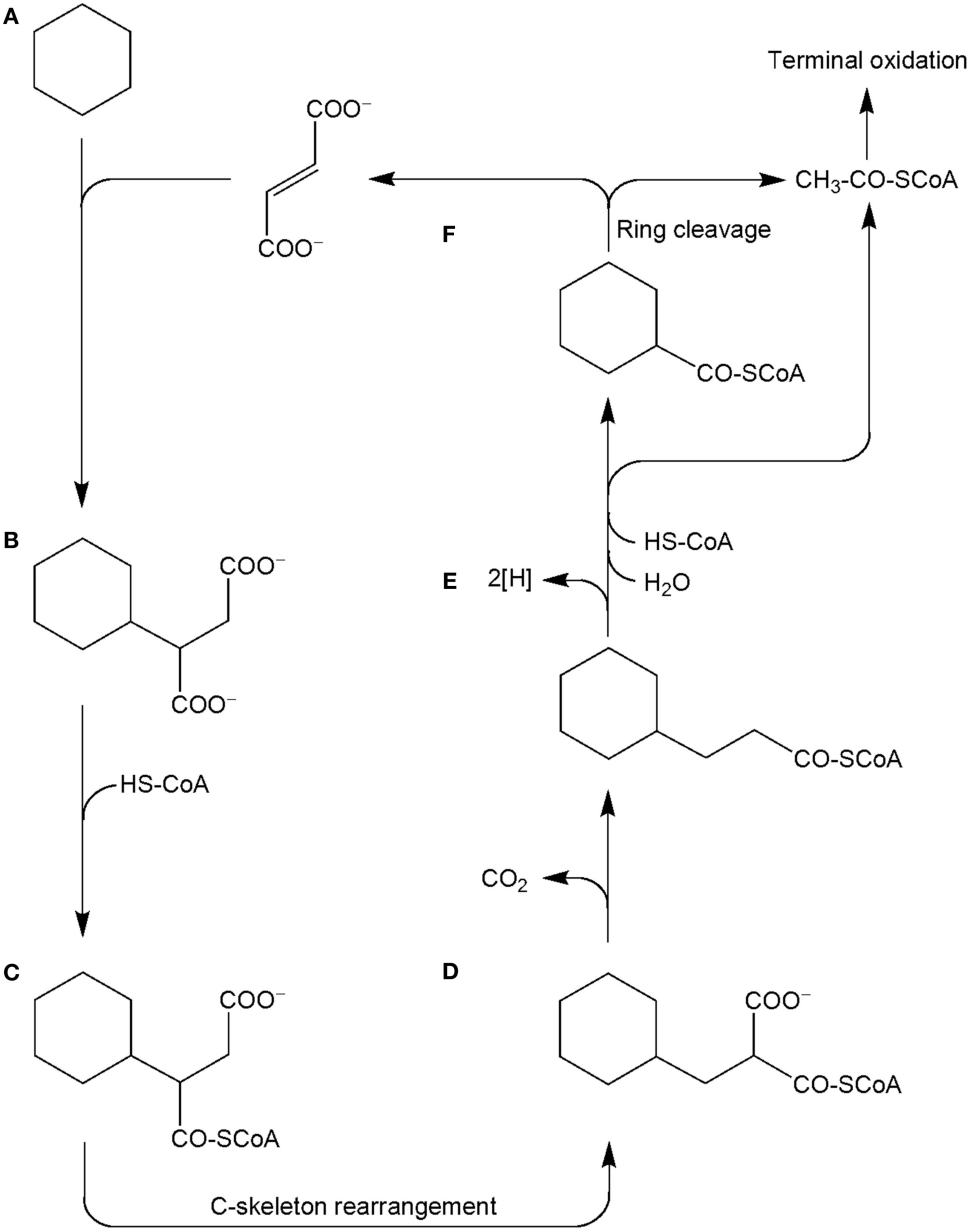
**Proposed pathway for the anaerobic degradation of cyclohexane by the enrichment culture Cyhx28-EdB, based on the detected metabolites (marked in bold-face)**. Cyclohexane **(A)** is activated by addition to fumarate, to yield cyclohexylsuccinate (**B**; detected in culture extracts). Cyclohexylsuccinate is further metabolized by activation to cyclohexylsuccinyl-CoA (**C**), C-skeleton rearrangement to (cyclohexylmethyl)malonyl-CoA (**D**) and decarboxylation to 3-cyclohexylpropionyl-CoA (**E**; detected as metabolite). β-Oxidation of cyclohexylpropionyl-CoA may explain the formation of cyclohexanecarboxyl-CoA **(F)**. Further oxidation and ring cleavage leads to acetyl-CoA, which is subjected to terminal oxidation and could also be used for the regeneration of fumarate.

Other metabolites detected in extracts of the culture Cyhx28-EdB grown with cyclohexane were identified based on the mass spectra of their corresponding methyl esters and comparison with standards as 3-cyclohexylpropionic acid and cyclohexanecarboxylic acid (Figure 6). The detection of these metabolites provides evidence that cyclohexylsuccinate is further degraded by ligation to coenzyme A, yielding cyclohexylsuccinyl-CoA, carbon-skeleton rearrangement and decarboxylation, yielding 3-cyclohexylpropionyl-CoA (detected as 3-cyclohexylpropionate), i.e., analogous to the pathway proposed for the degradation of *n*-alkanes (Wilkes et al., 2002) (Figure 7). β-Oxidation of 3-cyclohexylpropionyl-CoA yields cyclohexanecarboxyl-CoA (detected as cyclohexanecarboxylate) and acetyl-CoA. Further β-oxidation of cyclohexanecarboxyl-CoA would lead to ring cleavage, yielding pimelyl-CoA, and presumably malonyl-CoA and two acetyl-CoA. Alternatively, pimelyl-CoA could be further degraded via glutaryl-CoA and glutaconyl-CoA, yielding three acetyl-CoA, as proposed for the degradation of pimelate by denitrifying bacteria (Gallus and Schink, 1994). None of these proposed pathways yields fumarate (or succinate), which is essential for the activation of cyclohexane. We speculate that fumarate is synthesized from acetyl-CoA, via carboxylation to pyruvate and oxaloacetate, as previously proposed (Fuchs et al., 1978). Alternatively, fumarate may be regenerated via the propionyl-CoA pathway, through methylmalonyl-CoA and succinyl-CoA (Textor et al., 1997). Propionyl-CoA, which is most likely not a direct product of the cyclohexane degradation pathway, may be generated by other cellular processes, for example the β-oxidation of odd fatty acids. The acetyl-CoA could be further degraded to CO_2_ by the reverse Wood-Ljungdhal pathway, most common in complete-oxidizing sulfate-reducing bacteria (Schauder et al., 1986), as proposed or demonstrated for other alkane-degrading sulfate-reducing bacteria (e.g., Aeckersberg et al., 1991; Callaghan et al., 2012).

## Conclusions

We report here for the first time the complete degradation of cyclohexane under sulfate-reducing conditions. The enrichment culture obtained from intertidal hydrocarbon-contaminated marine sediments was dominated by a single phylotype affiliated with the *Desulfosarcina-Desulfococcus* cluster of the Deltaproteobacteria. Due to its abundance in the enrichment culture, we propose that this phylotype was responsible for the degradation of cyclohexane. Substrate tests with other hydrocarbons, corroborated with hybridization with sequence-specific oligonucleotide probes suggest that the dominant phylotype has a remarkable substrate range, being able to degrade both cyclic and *n*-alkanes, including the gaseous alkane *n*-butane. This is the first report of a sulfate-reducing bacterium from intertidal marine sediments being able to degrade gaseous alkanes. The current findings further expand our knowledge on the substrate range of *Desulfosarcina-Desulfococcus* affiliated bacteria, which are often found to be highly abundant in organic matter-rich marine sediments, including intertidal and arctic sediments (Ravenschlag et al., 2000; Llobet-Brossa et al., 2002), and hydrocarbon seep sites (e.g., Orcutt et al., 2010). The abundance of these microorganisms in such environments could be at least in part explained by their ability to degrade hydrocarbons, including cyclic and *n*-alkanes. Degradation of cyclohexane was initiated by addition to fumarate yielding cyclohexylsuccinate, a mechanism of activation commonly employed for the anaerobic degradation of *n*-alkanes. We also identified 3-cyclohexylpropionate, a metabolite which may enter the fatty acid synthesis cycle yielding odd ω-cyclohexyl-substituted fatty acids, as we previously demonstrated with a nitrate-reducing enrichment culture (Musat et al., 2010). Such fatty acids were often found in crude oil samples, with higher concentrations in crude oils affected by a medium to high level of biodegradation (Rodrigues et al., 2005). This suggests that microorganisms as those identified in the present study may be involved in the *in situ* biodegradation of crude oils under reservoir conditions or in formation waters.

### Conflict of interest statement

The authors declare that the research was conducted in the absence of any commercial or financial relationships that could be construed as a potential conflict of interest.
