# Supplementary material for: Anaerobic degradation of cyclohexane by sulfate-reducing bacteria from hydrocarbon-contaminated marine sediments
Source: Front Microbiol. 2015 Feb 20;6:116. doi: 10.3389/fmicb.2015.00116 (PMC4352924; doi:10.3389/fmicb.2015.00116)
Supplement: Supplementary file 1 [file DataSheet1.DOC]

**Anaerobic degradation of cyclohexane by sulfate**-**reducing bacteria from hydrocarbon-contaminated marine sediments**

**Ulrike Jaekel^1^, Johannes Zedelius^1^, Heinz Wilkes^2^, and Florin Musat^1,3*^**

^1^Department of Microbiology, Max Planck Institute for Marine Microbiology, Bremen, Germany

^2^Section 4.3: Organic Geochemistry, Helmholtz Centre Potsdam GFZ German Research Centre for Geosciences, Potsdam, Germany

^3^Present address: Department of Isotope Biogeochemistry, Helmholtz Centre for Environmental Research – UFZ, Leipzig, Germany

***Correspondence**: Florin Musat, Department of Isotope Biogeochemistry, Helmholtz Centre for Environmental Research – UFZ, Permoserstr. 15, 04318 Leipzig, Germany

florin.musat@ufz.de

**Keywords: cyclohexane, anaerobic, marine sediments, sulfate-reducing bacteria, Desulfosarcina**

**Supplementary information**

**
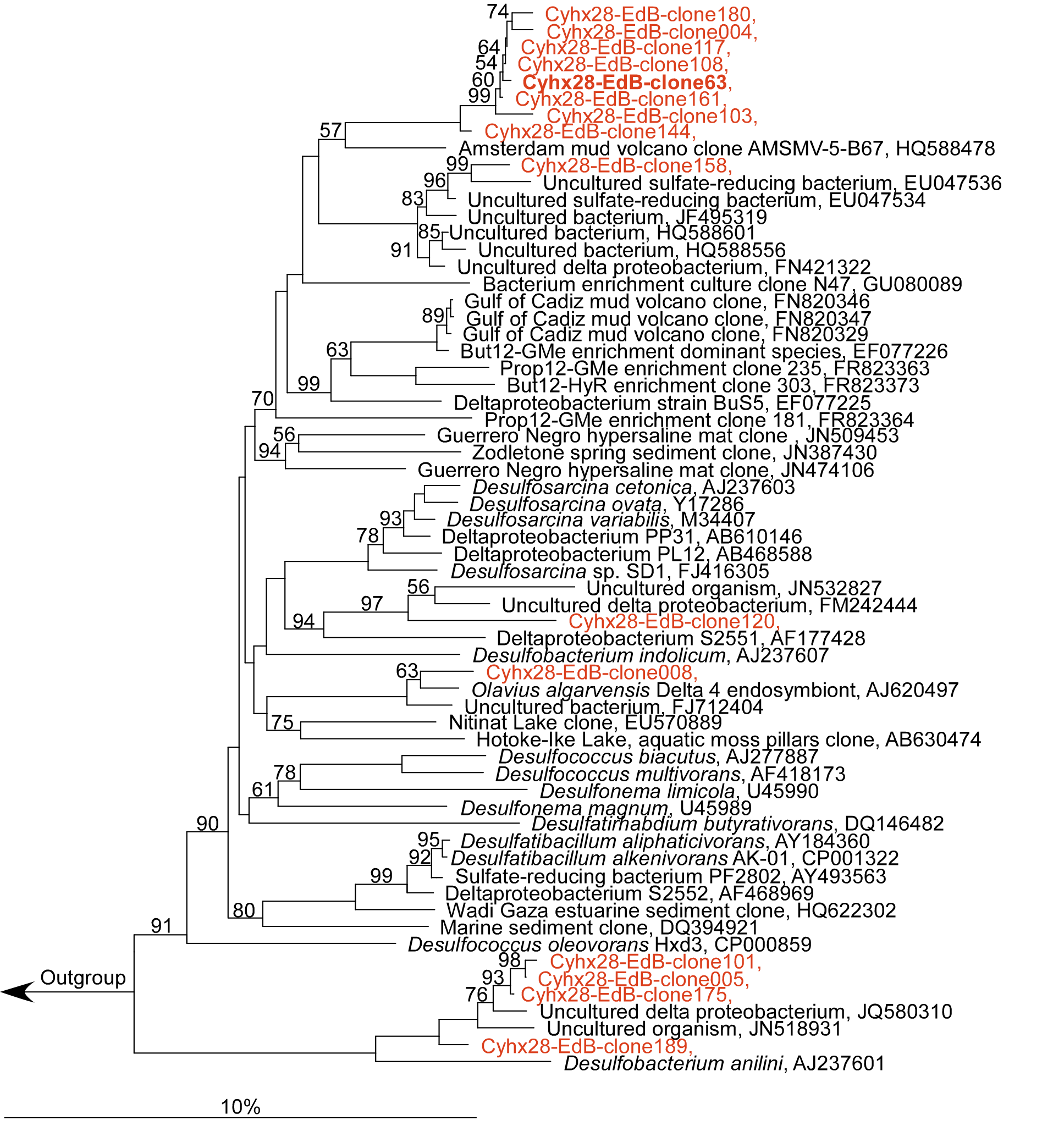
**

**Fig. SI1.** Phylogenetic relationship of the phylotypes affiliated with the Deltaproteobacteria in the enrichment culture Cyhx28-EdB (marked in red font). The dominant phylotype, Cyhx28-EdB-clone63 is marked in bold-face. The phylogenetic tree was calculated in ARB by neighbor-joining, using only nearly full-length sequences (> 1300 nt), with application of different sets of filters. The numbers next to nodes indicate bootstrap values higher than 50%. The scale bar represents 10% estimated sequence divergence.


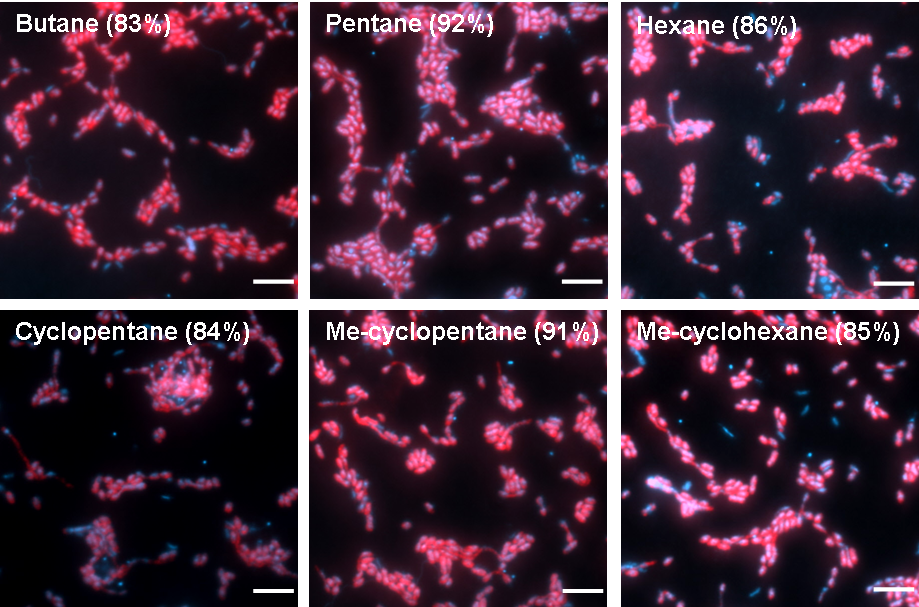


**Fig. SI2.** Whole-cell hybridization (CARD-FISH) of cells from the positive substrate-response tests with the sequence-specific probe Cyhx28-EdB_152, showing the dominance of the phylotype Cyhx28-EdB-clone63 in all samples. The images show an overlay of probe (red) and DAPI (blue) signals. Numbers on each image indicate the abundance, as percent of the total cell number, of the phylotype Cyhx28-EdB-clone63. Scale bars = 5 μm.

**Fig. SI3.** Mass spectra of the cyclohexylsuccinic acid dimethyl ester derived from the enrichment culture Cyhx28-EdB grown on cyclohexane. The mass spectra and GC retention time were identical to those of an authentic standard and with the mass spectra detected in a cyclohexane-degrading enrichment culture (Musat et al., 2010).

**Reference**

Musat, F., Wilkes, H., Behrends, A., Woebken, D., and Widdel, F. (2010). Microbial nitrate-dependent cyclohexane degradation coupled with anaerobic ammonium oxidation*. ISME* J 4, 1290-1301. doi: 10.1038/ismej.2010.50.
